# Phenotype Variations in a Family with Various Rearrangements in the Locus of the *SHOX* Gene

**DOI:** 10.3390/ijms27031580

**Published:** 2026-02-05

**Authors:** Tatiana S. Beskorovainaya, Tatiana V. Markova, Aleksander V. Polyakov, Olga A. Shchagina, Vladimir M. Kenis

**Affiliations:** 1 Research Centre for Medical Genetics, Moskvorechie Str., 1, 115522 Moscow, Russia; t-kovalevskaya@yandex.ru (T.S.B.); markova@med-gen.ru (T.V.M.); apol@dnalab.ru (A.V.P.); 2 H. Turner National Medical Research Center for Children’s Orthopedics and Trauma Surgery, Parkovaya Str., 64–68, 196603 Saint-Petersburg, Russia; kenis@mail.ru

**Keywords:** *SHOX* gene, LWD, PAR1, CNE, *CYP26C1* gene, deletion, duplication

## Abstract

The *SHOX* gene is located on both sex chromosomes, X and Y, within the pseudoautosomal region 1 (PAR1). Gross deletions at the SHOX locus lead to protein insufficiency and are manifested by growth disorders such as Leri-Weill dyschondrosteosis (LWD), Langer mesomelic dysplasia (LMD), and idiopathic short stature (ISS). In cases of the *SHOX* gene duplication, the phenotype may range from tall to short stature and LWD. This study describes a family with various SHOX locus alterations and diverse phenotypic manifestations. The proband inherited both deletion and duplication in the SHOX locus from her parents and shows typical features of LWD. The proband’s father carries *SHOX* gene deletion and displays Madelung’s deformity but normal height. The proband’s mother has *SHOX* gene duplication without any abnormalities in phenotype. One of the proband’s sons inherited deletion, while the other inherited duplication of the gene. Some family members also have the c.845_851dup variant in the *CYP26C1* gene, previously described as a modifier of the *SHOX* gene. It is difficult to assess its effect. At present, it is not possible to predict the future phenotype of the proband’s children due to the high phenotypic variability associated with SHOX locus alterations.

## 1. Introduction

Molecular defects of the *SHOX* gene, such as gross deletions, duplications, and pathogenic point variants, lead to reduced gene expression and insufficiency of the protein. These alterations result in phenotypes typically associated with short stature, including Leri-Weill dyschondrosteosis (LWD; MIM #127300) [1], Langer mesomelic dysplasia (LMD; OMIM #249700) [2,3], and idiopathic short stature (ISS; MIM #300582) [4].

LWD is a dominant hereditary disorder characterized by short stature, mesomelic limb shortening, and Madelung’s deformity of the forearms. It typically manifests in late childhood and frequently leads to significant functional impairments and chronic wrist pain. LMD is a more severe form of skeletal dysplasia, resulting from biallelic pathogenic variants in the *SHOX* gene [3,5,6]. ISS is diagnosed in individuals with height below −2 standard deviation score (SDS) without other alterations.

Pathogenic variants in the SHOX locus occur with a frequency of approximately 1 per 1000 newborns [4,7]. Heterozygous mutations in the *SHOX* gene and/or its regulatory elements are detected in 1.5% to 15% of children with ISS [8] and in 60% to 90% of patients with Leri-Weill dyschondrosteosis (LWD) [4,9,10,11].

Among the genetic alterations in the *SHOX* gene, 70–80% are gross deletions, 2–6% are partial deletions, and 20–25% are point variants [12,13,14]. Moreover, duplications of the SHOX locus have been described in patients with LWD and ISS [15].

The *SHOX* gene is located on both sex chromosomes (Xp22.33 and Yp11.2) within pseudoautosomal region 1 (PAR1), which extends to ~2.7 Mb. Each gonosome in this locus contains a homologous DNA sequence. This region escapes X-chromosome inactivation (XCI), and two functional copies of genes from this locus are required to provide normal function. The *SHOX* gene consists of seven exons (1–6a, b), and its transcripts are represented by a long isoform, SHOXa (NM_000451.4, 7934 bp, protein: 292 amino acids), encoded by exons 1–5 and 6a, and a short isoform, SHOXb (NM_006883.2, 1951 bp, 225 amino acids), which includes an alternative 3′-terminal exon 6b [16].

The *SHOX* gene is expressed in osteoblasts, bone marrow fibroblasts, and hypertrophic chondrocytes of the human embryo from the second month of gestation, as well as in the growth plate of long bones in the postnatal period [17]. The SHOX protein acts as a transcriptional activator, regulating chondrocyte proliferation, differentiation, and apoptosis by modulating the expression of several genes, including *SOX5*, *SOX6*, *SOX9*, *FGFR3*, *NPR2*, and *NPPB* [18,19,20,21,22].

The transcription of the *SHOX* gene depends on its interaction with 5′ and 3′ regulatory elements. It has been established that the *SHOX* gene is surrounded by conserved non-coding DNA elements (CNEs) that act as enhancers or repressors (https://www.ncbi.nlm.nih.gov/gdv/browser/gene/?id=108410392, accessed on 28 November 2025). Three CNEs with regulatory function are located upstream of the gene [hg19]: CNE-5 (chrX:398,357–398,950), CNE-3 (chrX:460,279–460,664), and CNE-2 (chrX:516,610–517,229) [23]. Five regulatory CNEs with transcriptional activity are located downstream of the gene [hg19]: CNE4 (chrX:714,084–714,753), CNE5 (chrX:750,824–751,850), CNE7 (chrX:780,700–781,220), CNE8 (chrX:811,550–812,300), and CNE9 (chrX:834,740–835,572) [24,25,26,27]. Additionally, two more regions with regulatory activity have been described: ZED (zeugopodal enhancer downstream of SHOX), located at chrX:827,128–827,691 [28], and a potential regulatory element located at chrX:970,000 [29,30]. Another three downstream elements—CNE2, CNE3, and CNE6, as well as upstream CNE-4—do not affect *SHOX* transcription [28].

In case of deletion or pathogenic point variant in the *SHOX* gene, the phenotype is caused by haploinsufficiency of the encoded protein. In case of *SHOX* duplication, the phenotype is difficult to predict. It may range from tall stature to short stature, LWD, or congenital clubfoot. The severity of clinical manifestations is probably caused by the size of duplication and the location of the additional copy. Moreover, the phenotypic features vary even within the same family, despite the high penetrance of pathogenic variants [9,31,32,33]. In addition, the development of pathology may be caused by deletions and duplications that do not affect the *SHOX* gene itself but only its regulatory elements [15,30,34,35,36]. This demonstrates the significant impact of the physical organization of the regulatory region on *SHOX* gene transcription. It is a complex challenge to determine whether duplications in the *SHOX* regulatory region are pathogenic since the mechanism of pathogenicity is not obvious.

The wide variability of phenotypes caused by defects in the SHOX locus and penetrance may also be due to the pathogenic variant in the modifiers of the *SHOX* gene. Thus, it was shown that the presence of pathogenic variants both in the *SHOX* and *CYP26C1* genes lead to more severe phenotypes when compared to patients carrying only the *SHOX* gene variant [37]. A similar aggravating effect on the phenotype of individuals with SHOX deficiency has been demonstrated for additional genetic alterations in some other genes [38]. It is also possible that not all regulatory elements of the *SHOX* gene have been identified yet [39].

This study describes a family with diverse phenotypes resulting from complex rearrangements of the *SHOX* gene locus.

## 2. Results

### 2.1. Case Presentation

#### 2.1.1. Family Pedigree

**Figure 1:**
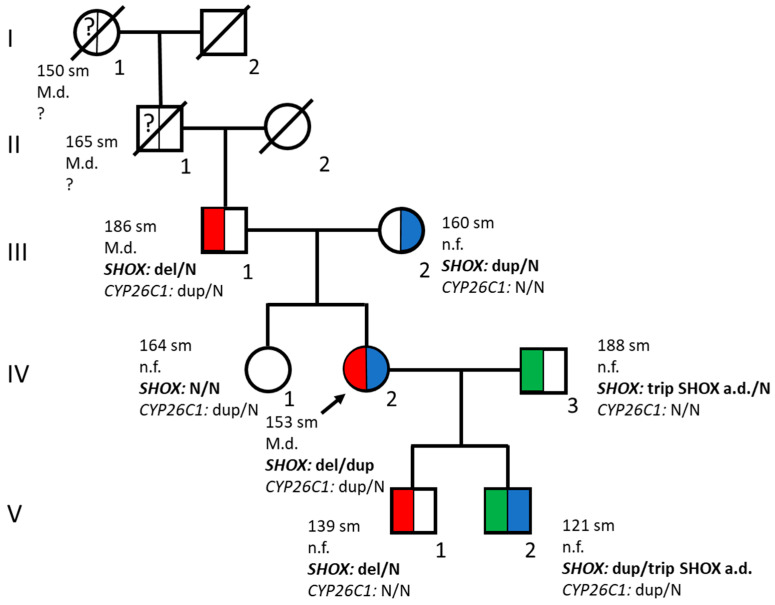
Pedigree of the family with various rearrangements of the SHOX locus (I–V, 1–3). M.d.—Madelung’s deformity, n.f.—normal forearms, *SHOX* del—deletion of the *SHOX* gene (red), *SHOX* dup—duplication of the *SHOX* gene (blue), *SHOX* trip SHOX a.d. (green)—triplication of a potential regulatory region (area down) within the SHOX locus, *CYP26C1* dup—pathogenic variant of c.845_851dup in the *CYP26C1* gene, N—normal alleles in the *SHOX* (white) and *CYP26C1* genes, ?, unknown. Circle—woman, square—man.

In the current study, genetic analysis was performed on patients from a family in which clinical examination revealed short stature and/or various skeletal deformities, and a family history of similar pathology was reported (Figure 1). The biological kinship of all examined family members was confirmed through molecular genetic methods.

#### 2.1.2. Proband’s Medical History

**Figure 2:**
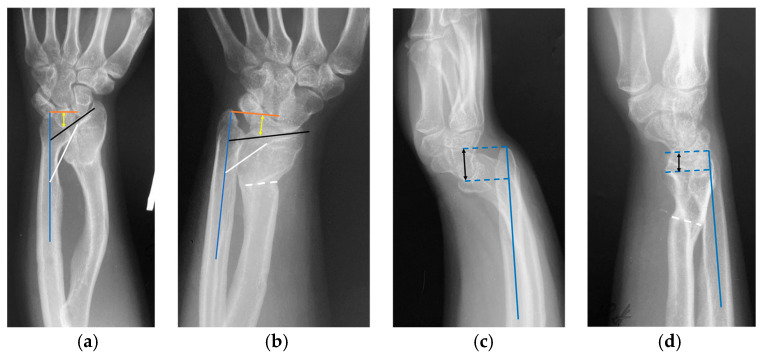
Radiographs of the proband’s wrist joint before and after surgical treatment. (**a**,**b**) posteroanterior radiographs of the patient’s wrist before (**a**) and after (**b**) surgery: ulnar tilt—the acute angle between the longitudinal axis of the ulna (blue line) and the line tangential to the proximal surfaces of the scaphoid and lunate (black line) changed from 47° to 78°; lunate fossa angle—the acute angle between the longitudinal axis of the ulna and the line across the lunate fossa of the radius (white line) changed from 30° to 55°; lunate subsidence—the distance between the most proximal point of the lunate and the line perpendicular to the longitudinal axis of the ulna (red line) and through its distal articular surface (yellow arrow) changed from 15 to 9 mm. The osteotomy level—white dotted lines. (**c**,**d**) lateral radiographs of the patient’s wrist before (**c**) and after (**d**) surgery: palmar carpal displacement—the distance between the longitudinal axis of the ulna and the most palmar point on the surface of the lunate or capitate (black arrow) changed from 18 to 10 mm. The osteotomy level—white dotted lines.

**Figure 3:**
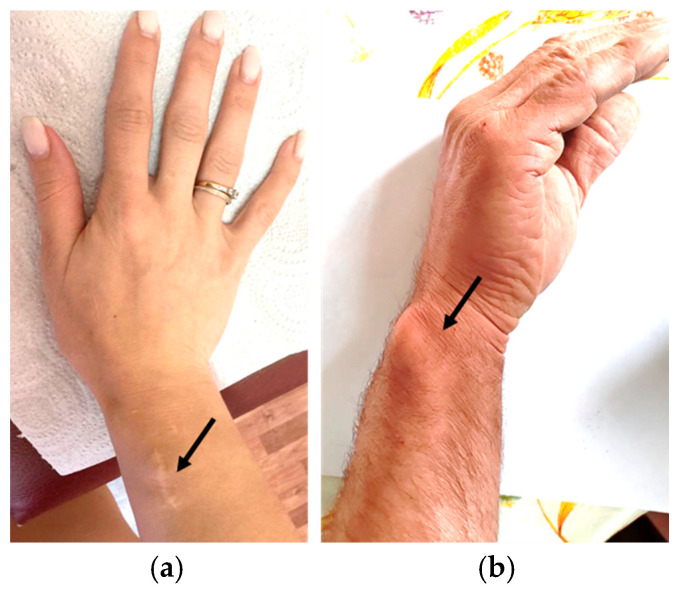
Condition of the wrist after surgical correction of the radius in the proband IV.2 (**a**) and Madelung’s deformity of the wrist in the proband’s father III.1 (**b**) (indicated by black arrows).

The proband is a 31-year-old woman (IV.2). Phenotype: height—153 cm (SDS = −1.5), weight—46 kg, disproportionate body build, mesomelic shortening of the limbs, Madelung’s deformity of the forearms and wrists, and the varus deformity of the tibia. Preliminary diagnosis: LWD. Medical history: since the age of 13, shortening of the limbs and deformity of the forearms have been observed (Figure 2a,c). At the age of 18, the patient was referred to orthopedic surgeons with obvious ‘bayonet-like’ wrist deformity, ulnar deviation of the hands, prominent ulnar head, impaired forearm rotation, and wrist pain. After discussion on surgical options (correction and lengthening with external fixators, single-stage radial corrective osteotomy, and ulnar shortening or isolated radial corrective osteotomy), she made the choice in favor of only radial corrective osteotomy (despite severe deformity and forearm bones’ length discrepancy). The patient was satisfied with the final functional and cosmetic results (Figure 3a). Radiographic parameters improved significantly (Figure 2b,d).

#### 2.1.3. Family History

The proband’s father, aged 57 (III.1), is 186 cm tall (SDS = +1.7), has an asthenic body type and Madelung’s deformity of the wrists (Figure 3b). The proband’s paternal grandfather (II.1, height 165 cm, SDS = −1.46) and paternal great-grandmother (I.1, height 150 cm, SDS = −2.0) exhibited limb shortening and Madelung’s deformity in the wrists. The proband’s mother, aged 55 (III.2), is 160 cm tall (SDS = −0.3) and has a stocky body build.

The elder son of the proband (V.1), initial examination at the age of 5: height—113 cm (SDS = +0.85), proportional body build, and mild pectus excavatum. Second examination at the age of 9: height—139 cm (SDS = +0.7), asthenic body build, planovalgus deformity of the feet, cubitus valgus, prominent scapulae, and mild myopia (Vis. OD, OS 1.0 with correction –1.5 D).

The younger son of the proband (V.2), initial examination at the age of 2: height—88 cm (SDS = +0.4), proportional body build, and congenital pectus carinatum. Second examination at the age of 6: height—121 cm (SDS = +0.6), asthenic body build, planovalgus deformity of the feet, cubitus valgus, joint hypermobility, and pectus carinatum.

The husband of the proband (IV.3), aged 30: height 188 cm (SDS = +2), normal body build, and no visible phenotypic alterations.

The sister of the proband (IV.1), aged 30: height 164 cm (SDS = +0.3), normal body build, and no visible phenotypic alterations.

### 2.2. Laboratory Test Results

**Figure 4:**
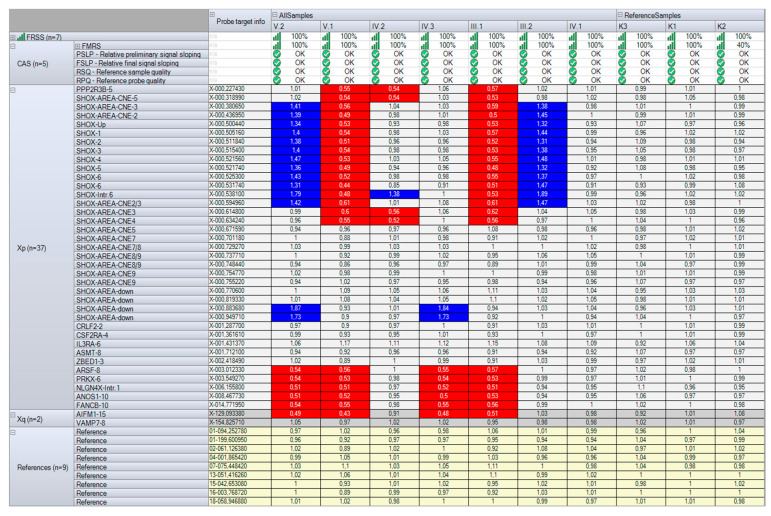
Results of the *SHOX* gene locus analysis through quantitative MLPA with the P018-G2 kit (MRC Holland, Amsterdam, The Netherlands). In individuals III.2 and V.2, heterozygous duplication was identified (colored blue). In individuals III.1 and V.1, heterozygous deletion was detected (colored red). In individual IV.2, a compound heterozygous combination of deletion and duplication was observed. In individuals IV.3 and V.2, triplication of uncertain clinical significance was identified in the SHOX-area-downstream region (colored blue). In individual IV.1, no quantitative alterations of the SHOX locus were detected. Individuals III.1, IV.3, V.1, and V.2 are male patients; individuals III.2, IV.1, and IV.2 are female patients; control samples K1, K2, and K3 are female.

The *SHOX* gene locus was analyzed using MLPA with the P018-G2 kit. All family members, aside from the proband’s sister, were found to have quantitative alterations in this locus (Figure 4).

In the proband’s father (III.1) and son (V.1), a heterozygous deletion was detected on the chromosome X: NC_000023.10:g.(?_307455)_(714247_?)del. This deletion includes the *SHOX* gene and regulatory elements located upstream of the gene—CNE-2, CNE-3, and CNE-5—as well as downstream of the gene—CNE4—and the inactive elements—CNE2 and CNE3.

In the proband’s mother (III.2) and the other son (V.2), a heterozygous duplication was identified on the chromosome X: NC_000023.10:g.(?_460638)_(675011_?)dup. This duplication includes the *SHOX* gene and regulatory elements upstream of the gene—CNE-2 and CNE-3—as well as inactive downstream elements—CNE2 and CNE3—which do not affect transcription. Additionally, in patients III.2 and V.2, an unexplained increase in the final ratio (FR) values was observed for the SHOX-Intr.6 probe (located in intron 6 of the SHOXb isoform), which is typical of triplication. Furthermore, in patient V.2 and his father (IV.3), triplication of uncertain clinical significance in heterozygous state was identified on the chromosome Y: NC_000024.9:g.(?_913682)_(979779_?)[3]. This triplication includes the SHOX-area-downstream region (enhancer area chrX:970,000 or chrY:920,000).

In proband IV.2, quantitative alterations detected in her parents (III.1 and III.2) were found in a compound heterozygous state: NC_000023.10:g.[(?_307455)_(714247_?)del];[(?_460638)_(675011_?)dup], both involving the *SHOX* gene and its regulatory regions. In the proband’s sister (IV.1), no quantitative changes in the SHOX locus were detected.

**Figure 5:**
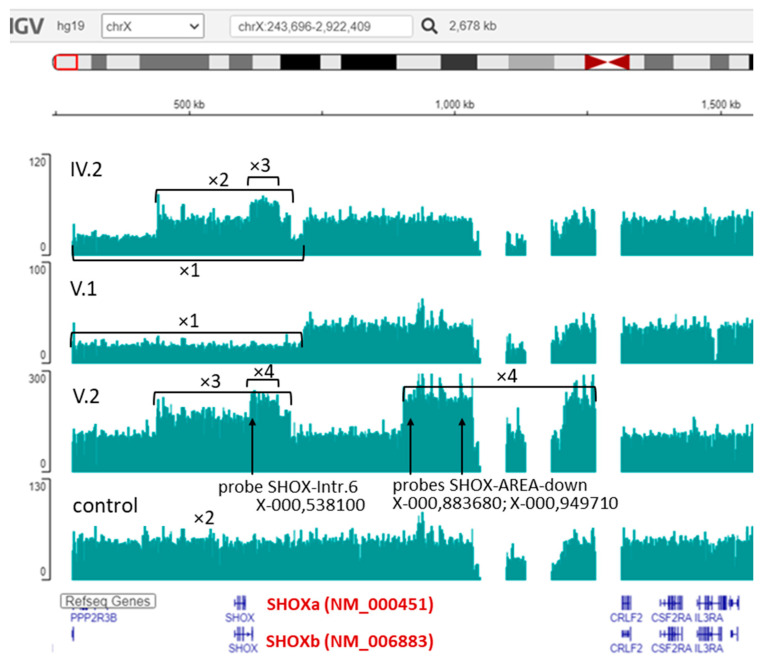
Results of WGS analysis of the *SHOX* gene locus in proband IV.2, her sons (V.1 and V.2), and a male control sample. The figure presents tracks displaying the coverage depth of the SHOX locus. The coverage profile confirms the results obtained by quantitative MLPA method. The designations ×1, ×2, ×3, and ×4 correspond to 1, 2, 3, and 4 copies of the locus, respectively. The arrows indicate the positions of the probes from the P018-G2 kit, for which triplication was detected through MLPA.

To determine the exact boundaries of the identified copy number variations in the SHOX locus, whole genome sequencing was carried out for the proband (IV.2) and her sons (V.1 and V.2). The coverage analysis results for the locus corresponded to the data obtained through the quantitative MLPA method (Figure 5).

**Figure 6:**
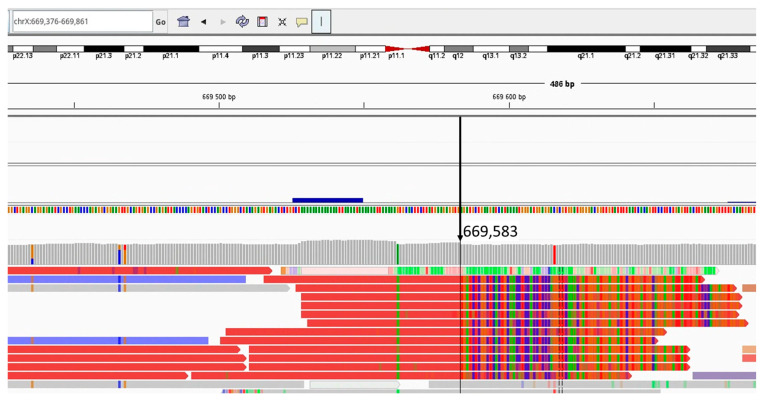
WGS analysis of the *SHOX* gene locus in patient V.2. The figure shows the bam-file track with chimeric reads from the region of the SHOX locus rearrangement. The arrow indicates the potential breakpoint at the genomic coordinate 669,583 [hg19].

Based on the WGS analysis, the refined boundaries of the deletion were determined as NC_000023.10:g.pter_717170del; of the duplication -NC_000023.10:g.[440234_615722dup;615723_669583[3];669584_691894dup]; and of the triplication—NC_000024.9:g.852160_1215000[3]. Chimeric reads produced from the regions of chromosomal rearrangements were also analyzed, and a search for the supposed breakpoint junctions was completed (Figure 6).

**Figure 7:**
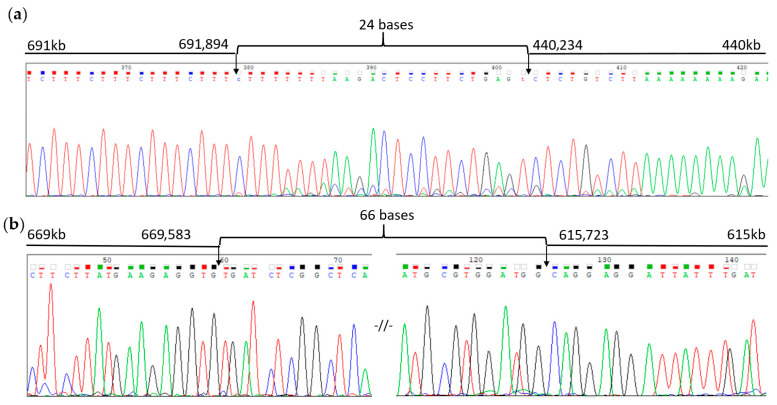
Sanger sequencing fragments of the SHOX locus in patient IV.2. The figure shows the junctions of DNA sequences: (**a**) from the 691 kb–440 kb region, with breakpoints at 691,894 and 440,234, connected by a 24-nucleotide fragment of unknown origin; (**b**) from the 669 kb–615 kb region, with breakpoints at 669,583 and 615,723, connected by a 66-nucleotide fragment of unknown origin, -//-—the sequence is partially not represented.

Subsequently, two supposed breakpoint regions—440,234–691,894 and 615,723–669,583—were validated through Sanger sequencing of PCR products obtained using primers flanking these regions. For the rearrangements 440,234–691,894 and 615,723–669,583, fragments of 550 bp and 250 bp, respectively, were obtained; further analysis confirmed the presence of tandem duplications (Figure 7).

The proband and her sons were also analyzed for point pathogenic variants in the genes responsible for growth disorders and limb deformities using the WGS method. The previously described pathogenic variant c.845_851dup (p.Gln284Hisfs*129) in the *CYP26C1* gene (NM_183374.3), described as a modifier of the *SHOX* gene, was found in proband IV.2 and the younger son (V.2), in a heterozygous state. In other family members, this variant was searched for through Sanger sequencing. It was also detected in proband’s father (III.1) and sister (IV.1) in a heterozygous state.

## 3. Discussion

Various alterations in the SHOX locus were identified in members of the examined family. In patients III.1 and V.1, the father and the son of the proband, respectively, a heterozygous deletion of the *SHOX* gene and adjacent regulatory elements were detected. The pathogenicity of the identified deletion is beyond doubt, seeing as it leads to haploinsufficiency of the *SHOX* gene. Pathogenic deletions involving only the gene itself, individual exons, deletions of regulatory elements alone, or the entire SHOX locus have been described in the literature [12,26,34,35]. According to ClinGen (clinicalgenome.org, accessed on 28 November 2025), the haploinsufficiency score for the *SHOX* gene is 3. This indicates that there is sufficient evidence for the pathogenicity of the gene deletion.

According to family history, the phenotypic manifestations among paternal relatives of the proband have some differences. While the examined patient III.1 only has Madelung’s deformity of the forearms (with normal height: 186 cm, SDS = +1.7), his father and paternal grandmother, who probably carry *SHOX* gene deletion as well, additionally exhibit limb shortening and short stature (165 cm, SDS = −1.46 and 150 cm, SDS = −2.0, respectively). This may reflect variable penetrance of the features, which is typical of pathogenic variants in this locus. At present, no symptoms have been observed in patient V.1. However, they may appear later, during the period of adolescence.

In patients III.2 and V.2, the mother and the other son of the proband, respectively, heterozygous duplication of the *SHOX* gene and adjacent regulatory elements was identified. According to ClinGen (clinicalgenome.org), the triplosensitivity score for the *SHOX* gene is 0. This indicates that duplications of the entire *SHOX* gene that can lead to protein overdosage are not currently considered clinically significant.

An additional copy of the *SHOX* gene and all its regulatory elements can lead to overexpression of the gene and tall stature, which is observed in individuals with an additional sex chromosome (47,XXX; 47,XXY; or 47,XYY) [40]. In case of partial duplication of the locus containing SHOX, predicting the pathogenicity of the identified insertion is particularly challenging. Although tall stature is sometimes observed with such duplications, *SHOX* gene duplication more often results in phenotypes associated with haploinsufficiency, manifesting as LWD, ISS, or clubfoot [41]. Duplications involving only the region of flanking regulatory elements have also been described as leading to SHOX haploinsufficiency [15,34,36,42]. Moreover, duplications of similar size do not always result in similar phenotypes. This can be explained by the structural organization of the rearranged locus and the extent to which the interaction between the *SHOX* gene and its regulatory elements is disrupted, thereby affecting the transcription of the gene.

**Figure 8:**
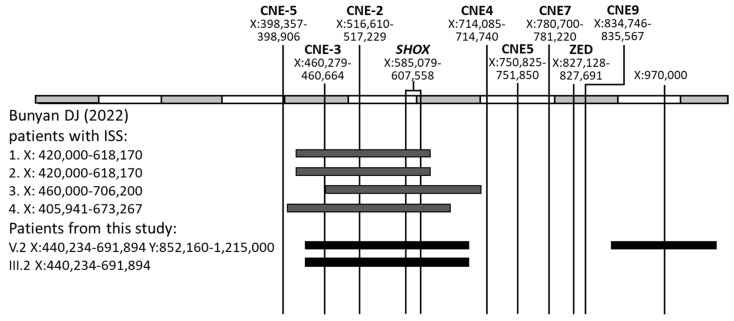
Previously described duplications [41] most similar in size to duplication (triplication) detected in this study.

**Figure 9:**
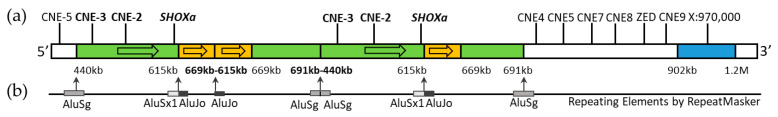
(**a**) Probable arrangement of the duplicated *SHOX* gene locus and its regulatory elements on the X chromosome in patients III.2, IV.2, and V.2. Positions 669 k–615 k and 691 k–440 k indicate the breakpoints confirmed by Sanger sequencing. The duplicated fragment is indicated in green, and the triplicated fragment is indicated in yellow. The arrows indicate the orientation of the inserts. The inserts have a forward direction, as well as the original locus. A fragment of the X chromosome for which there is a homologous sequence on the Y chromosome, triplicated in the patients IV.3 and V.2, is indicated in blue; (**b**) Repeating elements on the borders of the rearrangements involved in the process, according to the RepeatMasker track in UCSC genome browser (data last updated at UCSC: 20 February 2020).

Patients, previously described in the literature, with duplications most similar in size to the duplication identified in the current study, had ISS (Figure 8). In this study, the adult patient with duplication (III.2), demonstrates no significant clinical findings, and her height is normal. The unknown functional impact of this genetic variant is also indicated by the case of the proband IV.2, who, in addition to duplication, has deletion on the second X chromosome and has been diagnosed with LWD, a condition that manifests because of *SHOX* gene haploinsufficiency. Therefore, if both copies of the duplicated gene were fully active, the patient would have had a normal phenotype, seeing as duplication would compensate for haploinsufficiency caused by the deletion. Conversely, if the expression from both copies of the duplicated *SHOX* gene was critically impaired, the proband would likely have had a more severe phenotype, characteristic of LMD, which results from pathogenic variants in both copies of the gene on the X chromosomes. At the same time, in addition to Madelung’s deformity, the proband has a short stature compared to his father. Thus, this duplication may have a mild effect with a tendency to the severity of the phenotype. This can be explained by the genomic architecture of the locus, in which the insertion disrupts the function of one gene copy, while the other remains almost completely active, or both copies are transcribed with reduced efficiency due to the increased physical distance between the gene and some of its enhancers (Figure 9a). But it is premature to make a conclusion about the impact of the identified duplication based only on genomic data without additional functional tests. Unfortunately, the *SHOX* gene is not expressed in peripheral blood cells (https://gtexportal.org/home/gene/SHOX, accessed on 28 November 2025), so further studies on the gene dose are complicated. At this step of the study, the identified duplication should rather be considered as a variant of unknown significance (VUS).

It is also difficult to determine the effect of the detected g.615723_669583 triplication on the phenotype of patients III.2, IV.2, and V.2. Although this triplication itself does not contain regulatory elements, it can nevertheless physically distance them from the *SHOX* gene, thus affecting its expression.

The most likely mechanism of occurrence of the identified rearrangements is tandem duplication, in which the duplicated segment is inserted directly adjacent to the original locus. Tandem duplications account for up to 95% of gross duplications [43,44] and are caused by unequal crossing over [45]. The likelihood of unequal crossing over increases with a number of repetitive sequences flanking the duplicated region, which is also observed in this case (Figure 9b). The inserts detected in the current study have a forward direction, as well as the original locus, which is confirmed by Sanger sequencing of the breakpoints (Figure 7).

Patient V.2, who inherited a duplication from his grandmother (III.2), currently has normal height. However, it is known that phenotypes can vary even among individuals with identical pathogenic variants within the same family, and some features may manifest in later childhood or during puberty [33,46,47]. Moreover, this patient inherited from his father a triplication that includes an additional *SHOX* gene enhancer (chrY:920,000). The father (IV.3) is tall (188 cm, SDS = +2.0), probably due to this triplication. It was shown that deletion of this enhancer in the homozygous state led to clinical manifestations in the proband, but there were no abnormalities in heterozygous relatives [30,39]. Accordingly, it is likely that a 2-fold excess of this enhancer can increase the expression of the *SHOX* gene. Unfortunately, without a functional analysis, we can only hypothesize about the effect of this triplication and other identified inserts.

The SHOX locus investigated in the current study is located in the PAR1 region, which is present on both sex chromosomes (Xp22.33 and Yp11.2). Because genes in PARs escape XCI, mechanisms related to XCI cannot contribute to the phenotypic variability. However, it may occur due to the presence of other modifiers. For example, it was shown that pathogenic variants in heterozygous state in the *CYP26C1* gene, combined with pathogenic changes in the SHOX locus, lead to more severe phenotypes compared to phenotypes caused by a deficiency of only the *SHOX* gene [37]. CYP26C1 is an enzyme involved in the degradation of intracellular retinoic acid (RA). RA has been shown to play an important role during skeletogenesis [48]. Pathogenic variants in this gene can affect the *SHOX* gene expression and also independently lead to short stature in the absence of a SHOX locus defect [37,49]. In the current study, pathogenic variant c.845_851dup in the *CYP26C1* gene was found in some members of the studied family (Figure 1). This frameshift variant was previously described in homozygous and compound heterozygous state in relation to focal facial dermal dysplasia (FFDD) type IV [50]. It is also present in 0.2% of healthy individuals (https://gnomad.broadinstitute.org, accessed on 28 November 2025). The researchers supposed that height was not analyzed in patients with FFDD and control samples [49]. In our study, the correlation between the presence of this variant and height is questionable. The proband’s sister (IV.1), who has c.845_851dup in the heterozygous state, and proband’s father (III.1), who additionally has SHOX locus deletion, are of normal height. The short stature of the proband is probably due to the presence of the third variant—duplication on the second chromosome X. Thus, it is difficult to determine whether the combination of these two additional changes led to a more severe phenotype compared to the proband’s father, or only one of them made the main impact. It is also possible that the proband’s father and sister have mechanisms that compensate for high levels of RA by the gain of function variants in other genes involved in its metabolism. The combination of three variants—duplication of the SHOX locus, triplication of the *SHOX* gene enhancer, and c.845_851dup in the *CYP26C1* gene—is particularly interesting from the point of view of the effect on the growth of the proband’s son (V.2). At the moment, it is not possible to make a conclusion about the impact of the detected alternations on the *SHOX* gene expression, as well as about the phenotype of the child in the future. Such a variety of combinations of different variants in this family significantly complicates the interpretation of the observed phenotypes.

Although the X and Y chromosomes do not undergo recombination, the exchange of homologous regions is possible in the PARs. Consequently, in certain cases, inheritance of a pathogenic variant may not follow typical patterns of sex chromosome transmission. For example, a feature may be passed down from a grandmother through the father to a grandson, from a father both to sons and daughters, or from a father to only one of his sons or daughters. In the studied family, for instance, deletion was transmitted from the father (III.1) to only one of his daughters (IV.2), and a triplication of uncertain clinical significance in the *SHOX* gene enhancer was transmitted from the father (IV.3) to only one of his sons (V.2). Since these variants were identified in the blood cells of the fathers (III.1) and (IV.3), this phenomenon cannot be explained by germinal mosaicism. It is most likely associated with recombination between the X and Y chromosomes during gametogenesis.

## 4. Materials and Methods

In this study, family members from three generations with different phenotypes resulting from various rearrangements of the *SHOX* gene locus were clinically characterized and genetically tested. Informed consent for participation in the study was obtained from the patients and their legal representatives.

DNA extraction from peripheral blood samples collected in EDTA-containing tubes from examined family members was carried out using the Wizard^®^ Genomic DNA Purification Kit (Promega, Madison, MI, USA), according to the manufacturer’s protocol.

The copy number variation (CNV) analysis of the *SHOX* gene and the surrounding enhancer-containing regions was carried out using quantitative MLPA (multiplex ligation-dependent probe amplification) with the SALSA MLPA Probemix P018-G2 SHOX kit (MRC Holland, Amsterdam, The Netherlands), in accordance with the manufacturer’s protocol. The P018-G2 kit includes probes for each exon of the *SHOX* gene, as well as for each conserved non-coding element (CNE). Although the kit does not contain a probe for the potential regulatory element located at X:970,000, this element is flanked by probes at loci chrX:963,682–963,755 and chrX:1,029,698–1,029,779.

The separation of probe-derived fragments after ligation and PCR was carried out using a 3130 ABI Genetic Analyzer (Applied Biosystems, Waltham, MA, USA) with capillary electrophoresis. The data analysis was performed using the Coffalyser software version v.250317.1029 (MRC Holland, Amsterdam, The Netherlands) (https://support.mrcholland.com/downloads/files/coffalyser-net-installation-manual, accessed on 28 November 2025). After normalization with reference probes and control samples, the results were shown as a final ratio (FR), where the expected values are as follows:

- Normal copy number (two copies): FR = 0.80–1.20.
- Heterozygous deletion (one copy): FR = 0.40–0.65.
- Homozygous deletion (zero copies): FR = 0.
- Heterozygous duplication (three copies): FR = 1.30–1.65.
- Homozygous duplication/triplication (four copies): FR = 1.75–2.15.

The boundaries of rearrangements within the SHOX locus and the point nucleotide variants in the genomes were determined through whole genome sequencing (WGS) on a DNBSEQ-T7 genetic analyzer using paired-end 150 bp reads (PE150). Library preparation was carried out using a PCR-free protocol with enzymatic fragmentation (MGI).

Low-quality read ends and adapters were trimmed using cutadapt v.4.2 (https://github.com/marcelm/cutadapt, accessed on 28 November 2025). Read pair mapping was performed using BWA-mem v.0.7.17 (https://github.com/lh3/bwa, accessed on 28 November 2025) on the human reference genome hg19 and sorted with samtools v.1.16 (https://github.com/samtools/samtools, accessed on 28 November 2025). The hg19 was applied for the convenience of comparing the results obtained in this study with the substantial literature data in which this assembly was used. Duplicated reads in alignment were marked using MarkDuplicates from GATK v.4.3 (https://github.com/broadinstitute/gatk, accessed on 28 November 2025) with default parameters.

Variant calling was processed using the HaplotypeCaller algorithm from GATK. The identified genetic variants were designated in accordance with the HGVS nomenclature v.21.0.0 (https://hgvs-nomenclature.org, accessed on 28 November 2025). The population frequencies of these variants were obtained from the 1000 Genomes project and GnomAD v.2.1 (http://gnomad.broadinstitute.org/, accessed on 28 November 2025) data. The clinical (diagnostic) significance of variants was evaluated using the OMIM database (http://omim.org, accessed on 28 November 2025), Franklin Genoox (https://franklin.genoox.com/clinical, accessed on 28 November 2025), ClinVar (http://www.ncbi.nlm.nih.gov/clinvar/, accessed on 28 November 2025), and HGMD (https://digitalinsights.qiagen.com/products-overview/clinical-insights-portfolio/human-gene-mutation-database/, accessed on 28 November 2025).

The genome coverage profiles were generated using IGVtools and analyzed in the Integrative Genomics Viewer (IGV) (https://igv.org/, accessed on 28 November 2025).

The presumed breakpoint regions within the SHOX locus were validated by Sanger sequencing of PCR products obtained using primers complementary to the sequences flanking the breakpoints. Segregation in the family of the variant c.845_851dup in the *CYP26C1* gene was performed by Sanger sequencing using primers complementary to the exon 4 of this gene (NM_183374.3). Sanger sequencing was carried out using the 3130xl ABI Genetic Analyzer (Applied Biosystems, Waltham, MA, USA).

## 5. Conclusions

Genetic counseling for families with SHOX locus alterations is challenging due to the high phenotypic variability of the associated disorders. Taking into account the well-known age dependence of the severity of phenotypes associated with the SHOX locus, some clinical features may appear at a later age; therefore, long-term clinical monitoring of patients with changes in this locus is important. At present, it is not possible to predict the future phenotype and stature of the siblings V.1 and V.2. Regular examinations of children from this family are essential in order to assign timely treatment in case of growth delay and Madelung’s deformity, including the use of recombinant human growth hormone and orthopedic correction of wrist deformities.

## Data Availability

The data presented in this study are available on request from the corresponding author. Data are not publicly available due to privacy restrictions.

## Abbreviations

The following abbreviations were used in this manuscript:

LWD	Leri-Weill dyschondrosteosis
LMD	Langer mesomelic dysplasia
ISS	idiopathic short stature
PAR1	pseudoautosomal region 1
SDS	standard deviation score
CNE	conserved non-coding DNA element
M.d.	Madelung’s deformity
XCI	X-chromosome inactivation
RA	retinoic acid
MLPA	multiplex ligation-dependent probe amplification
FR	final ratio
WGS	whole genome sequencing
PCR	polymerase chain reaction
